# Correction: A Randomized Controlled Trial to Examine the Relationship Between Peer Mentoring for Physical Activity and Cardiometabolic Health

**DOI:** 10.5334/gh.1317

**Published:** 2024-03-21

**Authors:** Moradeke Bamgboye, David Adeyemi, Emmanuel Agaba, Susan Yilme, Clement A. Adebamowo, Sally N. Adebamowo

**Affiliations:** 1 Department of Physiology, University of Maryland School of Medicine, Baltimore, Maryland, United States; 2 Clinton Health Access Initiative, Abuja, Nigeria; 3 University of Jos, Jos, Nigeria; 4 Institute of Human Virology Nigeria, Abuja, Nigeria; 5 Department of Epidemiology and Public Health, University of Maryland School of Medicine, Baltimore, Maryland, United States; 6 University of Maryland Comprehensive Cancer Center, University of Maryland School of Medicine, Baltimore, Maryland, United States

**Keywords:** peer mentoring, physical activity, cardiometabolic health, randomized controlled trial

## Abstract

This article details a correction to: Bamgboye M, Adeyemi D, Agaba E, Yilme S, Adebamowo CA, Adebamowo, SN. A Randomized Controlled Trial to Examine the Relationship Between Peer Mentoring for Physical Activity and Cardiometabolic Health. *Global Heart*. 2023; 18(1): 53. DOI: https://doi.org/10.5334/gh.1268.

## Correction

Content of correction Some errors were identified in the original publication [1], primarily stemming from rounding inaccuracies or failure to update tables/figures after implementing changes.

– In Table 3, discrepancies were found where the Weekly METS was expressed in minutes, but the difference was expressed in hours. An updated Table 3 (provided below) presents all results consistently in minutes and hours; one corrected value is highlighted in bold.

**Table 3:** Participants Baseline and Follow-up Physical Activity, by Group.


PHYSICAL ACTIVITY	PEER MENTORED GROUP				CONTROL GROUP				*P*-VALUE^#^
	
	BASELINE	FOLLOW-UP	DIFFERENCE	*P*-VALUE*	BASELINE	FOLLOW-UP	DIFFERENCE	*P*-VALUE*	

Weekly METS (min)	9443.8 ± 4123.9	10750.3 ± 3751.4	+ 1306.5	0.006	11131.4 ± 4304.4	10366.3 ± 3052.9	–765.1	0.03	0.0005

Weekly METS (hr)	157.4 ± 68.7	179.2 ± 62.5	+ 21.8	0.006	185.5 ± 71.7	172.8 ± 50.9	–12.7	0.03	0.0005

Duration of moderate physical activity (min)	347.1 ± 458.9	776.0 ± 697.7	+ 428.9	<0.0001	480.2 ± 577.8	804.6 ± 800.4	+324.4	<0.001	0.30

Duration of moderate physical activity (hr)	5.8 ± 7.6	12.9 ± 11.6	+ 7.1	<0.0001	8.0 ± 9.63	13.4 ± 13.3	+5.4	<0.001	0.30

Duration of vigorous physical activity (min)	85.0 ± 213.2	169.3 ± 332.9	**+ 84.3**	0.003	120.3 ± 368.4	155.6 ± 442.1	+35.3	0.34	0.18

Duration of vigorous physical activity (hr)	1.4 ± 3.6	2.8 ± 5.5	+ 1.4	0.003	2.0 ± 6.1	2.6 ± 7.4	+0.6	0.34	0.18


Difference = difference between means. Min = minutes; hr = hours. *P*-value* = P-value comparing the mean values within each group, with a paired t-test. *P*-value^#^ = P-value comparing the difference in mean values between the groups, with a t-test.

– Table 5 contained two inaccuracies; specifically, the difference in the control group was –0.004 for waist-to-hip ratio (not –0.0009), and –0.2 for visceral fat (not +0.2). In the updated Table 5 (provided below) these corrected values are highlighted in bold.

**Table 5:** Impact of physical activity on participants cardiometabolic parameters.


CARDIOMETABOLIC PARAMETERS	PEER MENTORED GROUP				CONTROL GROUP				*P*-VALUE^#^
	
	BASELINE	FOLLOW-UP	DIFFERENCE	*P*-VALUE	BASELINE	FOLLOW-UP	DIFFERENCE	*P*-VALUE	

Weight, kg	89.5 ± 14.2	89.1 ± 14.5	– 0.4	0.24	88.6 ± 15.3	87.8 ± 14.7	– 0.8	0.07	0.42

BMI, kg/m^2^	33.2 ± 6.6	33.0 ± 6.6	– 0.2	0.23	31.9 ± 5.4	31.6 ± 5.3	– 0.3	0.07	0.48

Waist-Hip Ratio	0.927 ± 0.05	0.910 ± 0.07	–0.017	0.002	0.919±0.06	0.915±0.07	–**0.004**	0.83	0.014

Total body fat, %	41.3 ± 8.3	41.4 ± 9.2	+ 0.1	0.63	40.4 ± 9.4	39.6 ± 9.7	– 0.8	0.02	0.09

Visceral fat, %	11.5 ± 4.0	11.3 ± 3.6	– 0.2	0.55	11.3 ± 4.2	11.1 ± 3.8	**– 0.2**	0.93	0.67

Skeletal muscle, %	26.2 ± 4.7	26.3 ± 4.8	+ 0.1	0.84	26.5 ± 5.1	26.8 ± 5.3	+ 0.3	0.11	0.49

Cholesterol, mg/dl	204.1 ± 43.3	183.8 ± 38.8	– 20.3	<0.0001	207.2 ± 43.9	182.4 ± 40.4	– 24.8	<0.0001	0.29

Triglycerides, mg/dl	123.7 ± 59.8	101.1 ± 45.0	– 22.6	<0.0001	132.2 ± 77.2	102.4 ± 50.5	– 29.8	<0.0001	0.81

LDL-Chol	133.8 ± 41.0	113.3 ± 36.1	– 20.5	<0.0001	136.8 ± 39.7	113.0 ± 37.3	– 23.8	<0.0001	0.63

HDL-Chol	44.4 ± 9.9	50.1 ± 7.6	+ 5.7	<0.0001	44.2 ± 16.8	48.9 ± 8.3	+ 4.7	<0.0001	0.72


LDL = low-density lipoproteins. HDL = high-density lipoproteins. Chol = cholesterol. Difference = difference between means. *P*-value* = P-value comparing the mean values within each group, with a paired t-test. *P*-value^#^ = P-value comparing the difference in mean values between the groups, with a t-test.

– Figure 3 was revised to clarify the direction of change for each parameter.

**Figure 3:**
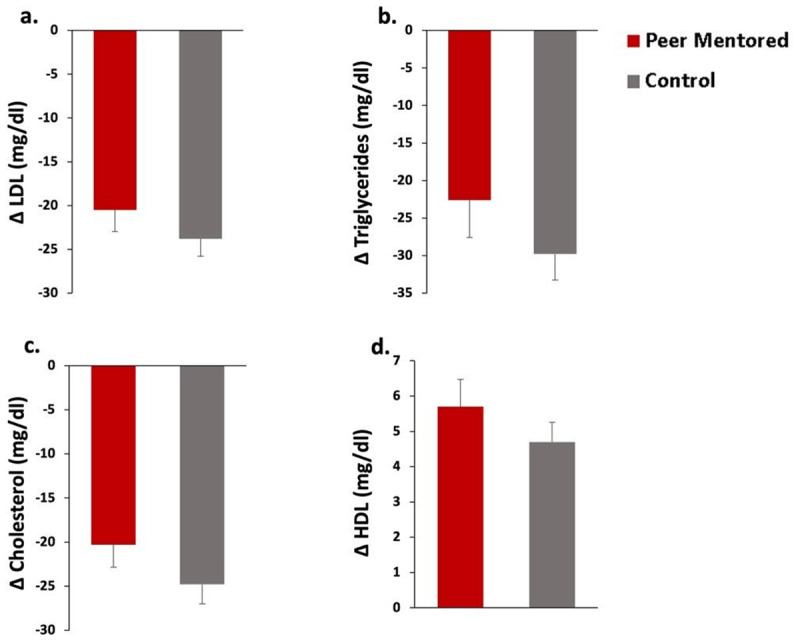
Change in cardiometabolic parameters by group. **a.** change in low-density lipoproteins (LDL). **b.** change in triglycerides. **c.** change in cholesterol. **d.** change in high-density lipoproteins (HDL).

We emphasize that due to the culture at the enrolment site, the observations are not dependent within the same office space.

These corrections do not change the overall results or conclusions.
